# Prevalence of multimorbidity in men of African descent with and without prostate cancer in Soweto, South Africa

**DOI:** 10.1371/journal.pone.0276050

**Published:** 2022-10-18

**Authors:** Witness Mapanga, Shane A. Norris, Ashleigh Craig, Yoanna Pumpalova, Oluwatosin A. Ayeni, Wenlong Carl Chen, Judith S. Jacobson, Alfred I. Neugut, Mazvita Muchengeti, Audrey Pentz, Sean Doherty, Shauli Minkowitz, Mohammed Haffejee, Tim Rebbeck, Maureen Joffe

**Affiliations:** 1 Noncommunicable Diseases Research Division, Wits Health Consortium (PTY) Ltd, Johannesburg, South Africa; 2 SAMRC/Wits Developmental Pathways to Health Research Unit, Department of Paediatrics, Faculty of the Health Sciences, University of the Witwatersrand, Johannesburg, South Africa; 3 SAMRC Common Epithelial Cancer Research Centre, Cape Town, South Africa; 4 Division of Medical Oncology, Department of Medicine, School of Clinical Medicine, Faculty of Health Sciences, University of the Witwatersrand, Johannesburg, South Africa; 5 Global Health Research Institute, School of Human Development and Health, University of Southampton, Southampton, United Kingdom; 6 Department of Medicine, Vagelos College of Physicians and Surgeons, Columbia University, New York, New York, United States of America; 7 National Cancer Registry, National Health Laboratory Service, Johannesburg, South Africa; 8 Sydney Brenner Institute for Molecular Bioscience, Faculty of Health Sciences, University of the Witwatersrand, Johannesburg, South Africa; 9 Herbert Irving Comprehensive Cancer Center, Vagelos College of Physicians and Surgeons, Columbia University, New York, New York, United States of America; 10 Department of Epidemiology, Mailman School of Public Health, Columbia University, New York, New York, United States of America; 11 Division of Urology, Department of Surgery, Faculty of Health Sciences, University of the Witwatersrand, Johannesburg, South Africa; 12 Dana Farber Cancer Institute, Boston, Massachusetts, United States of America; 13 Harvard T.H. Chan School of Public Health, Boston, Massachusetts, United States of America; The University of the West Indies, JAMAICA

## Abstract

**Objective:**

With increases in chronic disease, men with prostate cancer are likely to have at least one other chronic health condition. The burden and complexity of each additional chronic disease may complicate prostate cancer treatment and reduce survival. In this paper, we describe the frequency of multimorbid chronic diseases, HIV and depression among men in Soweto, South Africa (SA) with and without prostate cancer and determine whether the presence of multimorbid diseases is associated with metastatic and high-risk, non-metastatic prostate cancer.

**Methods:**

A population-based case-control study on prostate cancer was conducted among black men in Soweto. All participants completed a baseline survey on sociodemographics, lifestyle, and comorbid medical conditions. All participants completed a depression screening survey and HIV testing at enrolment. Blood pressure measurements and blood testing for fasting glucose, total cholesterol, and high-density lipoprotein were performed on a subset of randomly selected cases and controls. For men with prostate cancer, clinical T staging was assessed with the digital rectal examination, the diagnosis was confirmed with a biopsy and PSA levels were assessed at presentation. The metastatic staging was assessed by bone scans, and this was confirmed with PSMA PET scans, CT scans and X-rays, standard for our resource-constrained setting. Normal PSA scores were used as an inclusion criterion for controls.

**Results:**

Of the 2136 men (1095 with prostate cancer and 1041 controls) included in the analysis, 43.0% reported at least one chronic metabolic disease; 24.1% reported two metabolic diseases; 5.3% reported three metabolic diseases; and 0.3% reported four metabolic diseases. Men with prostate cancer were more likely to report a multimorbid chronic metabolic disease compared to controls (p<0.001) and more likely to test positive for HIV (p = 0.05). The majority of men (66.2%) reported at least one metabolic disease, tested negative for HIV and had a negative depression screen. The clinical characteristics of men with prostate cancer, were as follows: 396 (36.2%) had a Gleason score of 8 and above; 552 (51.3%) had a PSA score of >20ng/ml; 233 (21.7%) had confirmed metastatic prostate cancer at diagnosis. Older age was associated with metastatic prostate cancer (OR = 1.043 95% CI:1.02–1.07) and NCCN defined high-risk non-metastatic prostate cancer (OR = 1.03 95% CI:1.01–1.05), whilst being hypertensive was protective (OR = 0.63 95% CI:0.47–0.84 and OR = 0.55 95% CI:0.37–0.83) respectively for metastatic and high-risk, non-metastatic prostate cancer.

**Conclusion:**

The high prevalence of multimorbid metabolic diseases and HIV among men with prostate cancer represents a public health concern in South Africa. There is a need to effectively address multiple chronic diseases among men with prostate cancer by incorporating coordinated care models.

## Introduction

Although data from sub-Saharan Africa (SSA) is sparse, prostate cancer is estimated to be the leading cause of cancer morbidity and mortality among African men [1, 2]. Evidence from the beginning of the last decade estimates that in SSA, roughly 54 per 100,000 men were diagnosed with prostate cancer annually, whilst 20 per 100,000 died from prostate cancer, with some 57,048 deaths expected by 2030 [3, 4]. Recently, the International Agency for Research on Cancer (IARC) reported that prostate cancer is the leading cancer in terms of both incidence and mortality in men in SSA and the Caribbean and that incidence is expected to more than double in the next two decades [5]. Although prostate cancer mortality rates continue to decline in high-income global regions, incidence and mortality are rising rapidly in most of SSA [1].

Men of African descent have the highest rates of prostate cancer of any racial or ethnic group. In the US, the incidence and mortality rates are 71% and 210% higher respectively in African American men compared to Caucasian American men [6]. Despite the public health implications of these observations, the underlying cause of this disparity remains unresolved. In South Africa (SA), which is estimated to have the highest rates of both morbidity and mortality from prostate cancer in SSA, there is a lack of prioritisation of cancer research, prevention, treatment and overall management due to a multitude of factors such as lack of funding and the high burden of HIV and tuberculosis [7, 8].

The observed rapid rise in prostate cancer incidence in SA and across SSA may be due to an increased prevalence of other chronic diseases such as HIV-AIDS, diabetes, or obesity, or due to unhealthy lifestyle choices, including physical inactivity, diets lacking fruit and vegetables, smoking, alcohol intake and reproductive behaviours [7–9]. A population-based case-control study in Finland identified that middle-aged men diagnosed with metabolic syndrome were more likely to develop prostate cancer when compared to men without metabolic syndrome and that this risk was even greater in men who were overweight and obese [10]. Similar findings were also reported among African men from various countries including SA, where overall and central obesity was associated with an increased risk of prostate cancer [11]. The association between metabolic syndrome and prostate cancer has also been reported elsewhere [12–14]. Evidence generated across different populations has also indicated that prostate cancer might be a common malignancy among HIV-positive men [15]. However, there has been conflicting evidence as other studies indicate that HIV-positive men have a decreased risk of prostate cancer [16]. Therefore, in this study, we aim to describe the multimorbid health conditions among African men in SA with and without prostate cancer and to determine factors associated with prostate cancer stage at diagnosis. Such findings might inform the treatment and management of prostate cancer in SA so that multiple morbidities are also addressed in a holistic approach.

## Methods

### Study setting and data source

This prostate cancer case-control cohort study was set up and coordinated by the University of the Witwatersrand and based at the Chris Hani Baragwanath Academic Hospital (CHBAH), in Soweto, Johannesburg. CHBAH is one of seven African study sites participating in the Men of African Descent and Prostate Cancer (MADCaP) Consortium [17]. Men were recruited at urology and non-urology outpatient clinics between October 2016 and August 2020. CHBAH is the third-largest hospital in the world and a government tertiary hospital that predominantly serves the black urban community in southern and eastern Johannesburg. Most of the patients presenting at CHBAH are referred from both the public primary and secondary care facilities around Soweto, a mainly black urban community in southern Johannesburg.

### Recruitment of cases and control

#### Cases

Men of self-reported African descent aged 30 years and above who provided written consent and had a histologically confirmed new diagnosis of prostate cancer were included. Exclusion criteria were a previous cancer diagnosis or inability to provide written consent or answer questions.

#### Controls

Men of African descent, aged 30 years and above who had a normal PSA and provided written consent were recruited predominantly from the CHBAH Ophthalmic department. Controls matched cases for age (±5 years). Controls were selected from the same communities as the cases.

### Procedures

#### Interviews and staging

Face-to-face interviews were conducted by research investigators and trained study staff. Socio-demographic characteristics (age, education) and self-reported comorbidities (defined as being on chronic treatment for hypertension, diabetes, or hyperlipidaemia) were determined at the time of study enrolment. Depression was assessed using the Patient Health Questionnaire-9 (PHQ-9), which was completed at study enrolment. Diagnoses were all confirmed with a core biopsy of the prostate, with Gleason scores reported. PSA was routinely assessed at diagnosis and follow-up visits for surveillance of the disease. Cases were clinically T- staged by digital rectal examination, and metastatic disease was assessed by bone scans confirmed as indicated with prostate-specific membrane antigen (PSMA) PET scans, abdominal CT scans and X-rays, which is the standard for our resource-constrained public tertiary hospitals.

#### Blood measurements

The Randox Daytona Plus automated clinical chemistry analyser method was used to measure fasting plasma glucose and lipid profile (high-density lipoprotein (HDL) and total cholesterol (TC)) for randomly selected cases and their age-matched controls (laboratory patient subset). For the rest of the cohort, diagnosis of comorbid diabetes and hyperlipidaemia was self-reported, as described above. All men enrolled in the study underwent HIV testing using the enzyme-linked immunosorbent assay (ELISA).

#### Anthropometric measurements

Blood pressure (BP) was measured on seated participants for the laboratory patient subset, using the average of the 2^nd^ and 3^rd^ readings [18], according to the American Heart Association guidelines and recommendations [19]. For the remaining cohort, diagnosis of comorbid hypertension was self-reported, as described above. Height, waist and hip circumferences, and weight were measured in the complete cohort.

#### Multimorbidities

For the cohort in whom we measured blood pressure, we categorised men as hypertensive or normotensive using a cut-off of 140/90 mmHg. For the laboratory patient subset, we defined dyslipidaemia as a TC level of >6.21 mmol/L or HDL level of <1.19 mmol/L and defined impaired fasting glucose as a fasting glucose level of >5.5 mmol/L [20]. Patients were not required to be fasting for lipid profile lab measurements. Self-reported participants were accordingly assigned to these groups. Obesity for the cohort was determined by calculating the BMI from the weight and height of each participant using the WHO obesity cut-offs: a BMI of <18.5 kg/m2 was categorised as underweight, 18.5–24.9 kg/m2 as normal, 25–29.9 kg/ m2 as overweight, 30–39.9 kg/m2 as obese, and ≥40 kg/m2 as morbidly obese [21]. We further categorized the cohort by the number of comorbid metabolic diseases. The metabolic disease was defined as obesity (body mass index ≥30 kg/m2), dyslipidaemia (total cholesterol >6.21 mmol/L or self-reported dyslipidaemia on treatment), hypertension (measured blood pressure ≥140/90 mmHg or self-reported hypertension on treatment), or hyperglycaemia (fasting glucose >5.5 mmol/L).

#### Study outcome measurement

The primary outcome was the prostate cancer stage at diagnosis. Men with prostate cancer were categorised as having localised or metastatic disease using bone scan, confirmed PMSA PET, abdominal CT and X-rays. Men with the non-metastatic disease at diagnosis were further categorised into locally defined prostate cancer risk groups as per National Comprehensive Cancer Network (NCCN) guidelines [22]. The nonmetastatic low-risk disease was defined as a PSA level of less than 10 ng/ml, Gleason grade group 1 (Gleason score 3 + 3) and clinical stage T1‒T2a. Intermediate high-risk was defined as cT2b or cT2c, and/or has a Grade Group of 2 or 3 (Gleason score of 7) and/or a PSA level between 10 and 20 ng/ml. High risk was defined as stage cT3a, Gleason score of 8–10 independent of PSA values, or PSA of >20 ng/mL, and very high risk as cT3b, cT3c or T4 disease.

#### Statistical analysis

Statistical analyses were performed using Stata version 17 (Stata Corp Ltd, Texas, USA). The socio-demographic profile and comorbid disease profile in both cases and controls were analysed, described and reported using Pearson’s χ2 and Fisher’s exact tests for categorical variables and Student’s t-test for continuous variables. Metabolic disease frequencies within the cohort were also reported. Three stepwise multivariate models (logistic regression) were used to describe factors (socio-demographic characteristics and comorbidities) related to metastatic prostate cancer at diagnosis. The odds ratios were used to determine the strength of association in three models: stage and socio-demographic characteristics; stage and comorbidities; and stage and combined socio-demographic characteristics and comorbidities. A separate multivariable logistic regression model was used to examine the association of socio-demographic characteristics and comorbidities with high-risk non-metastatic prostate cancer defined as stage cT3a or higher, or Gleason score of 8–10 independent of PSA values, or PSA of >20 ng/mL.

#### Ethical approval and consent to participate

This study was approved by the University of the Witwatersrand Human Research Ethics Committee (Reference number: M150934). All participants provided written informed consent.

## Results

A total of 2136 men (1095 cases and 1041 controls) were analysed. The mean age of the total cohort was 65.1 years (standard deviation (SD) 8.9). Men with prostate cancer were significantly older than controls (67.0 (SD 8.0) vs 63.0 (SD 9.0) years; p<0.001), and more likely to be hypertensive (66.3% vs 48.7%, p<0.001). Men with prostate cancer were less likely to have completed secondary education compared to men without prostate cancer (37.4% vs 60.1%, p<0.001). There were no significant differences in BMI between the cases and controls (Table 1).

**Table 1:** Self-reported sociodemographic and multimorbidity in men with and without prostate cancer.

Variables	Total (N = 2136), n(%)	Men with prostate Cancer (n = 1095), n(%)	Men without prostate cancer (n = 1041), n(%)	p-value
**Sociodemographic**				
Age (years), mean (SD)	65.1(8.9)	67.0(8.0)	63.0(9.0)	**< .001**
**Level of education**				
Primary education and below	1100 (51.5)	685(62.6)	415(39.9)	**< .001**
Secondary and some tertiary education	1036 (48.5)	410(37.4)	626(60.1)	
**BMI (kg/m** ^**2**^ **)** ^a^				
(Underweight) <18.5	92 (4.3)	42 (3.8)	50 (4.8)	.538
(Normal) 18.5–24.9	831 (39.0)	419 (38.3)	412 (39.7)	
(Overweight) 25–29.9	701 (32.9)	364 (33.3)	337 (32.5)	
(Obese) ≥30	507 (23.8)	269 (24.6)	238 (23.0)	
**Self-reported hypertension on treatment** ^a^				
No	902 (42.3)	369(33.7)	533(51.3)	**< .001**
Yes	1231 (57.7)	725(66.3)	506(48.7)	
**Self-reported high blood sugar or diabetes on treatment** ^a^				
Yes	1755 (82.2)	183 (16.7)	196 (18.9)	0.440
No	379 (17.8)	911 (83.3)	844 (81.1)	

^a^The following had missing values: BMI (1 case plus 4 controls); Hypertension (1 case plus 2 controls); Diabetes (1 case plus 1 control)

Overall, 12% of men included in the analysis were HIV positive (n = 247) and men with prostate cancer were more likely to be HIV positive, compared to controls (14.0% vs 10%, p = 0.005). Men with prostate cancer were more likely to have a measured HDL > 1.3 mmol/L (p<0.001) when compared to men in the control group. There were no significant differences in measured waist circumference, fasting plasma glucose or rates of depression between the cases and controls (Table 2).

**Table 2:** Measured multimorbidity profile in men with and without prostate cancer.

Variables	Total n(%)	Men with prostate Cancer n(%)	Men without prostate cancer n(%)	p-value
**Waist circumference (cm), mean (SD)**^a^ **(N = 2136)**	94.4(12.6)	94.3(12.4)	94.5(12.8)	.620
**Communicable and non-communicable disease morbidities**				
**Dyslipidaemia (total cholesterol (TC) (N = 878)**				
TC≤6.21	875 (99.7)	670 (99.6)	205 (100.0)	.338
TC>6.21	3 (0.3)	3(0.4)	0(0.0)	
**Dyslipidaemia (High-density lipoprotein (HDL) in mmol/L (N = 872)**				
HDL≤1.3	473 (54.2)	338(50.6)	135(66.2)	**< .001**
HDL>1.3	399 (45.8)	330(49.4)	69(33.8)	
**Hypertension (N = 400)**				
No	178 (44.5)	85 (42.5)	93 (46.5)	**< .001**
Yes	222 (55.5)	115 (57.5)	107 (53.5)	
**Fasting glucose (mmol/L) (N = 406)**				
≤5.5	240 (59.1)	112(54.6)	128(63.7)	.050
>5.5	166 (40.9)	93(45.4)	73(36.3)	
**HIV status**^b^ **(N = 2054)**				
Positive and on treatment	247 (12.0)	147(14.0)	100(10.0)	.**005**
Negative	1807 (88.0)	905(86.0)	902(90.0)	
**Risk of depression (based on the PHQ-9 scale) (N = 2136)**				
Yes	4 (0.2)	0(0.00)	4(0.4)	.040
No	2,132 (99.8)	1,095(100.0)	1,037(99.6)	

^a^Waist circumference was measured for the whole cohort

^b^HIV status was measured for the whole cohort, with only 82 (43 cases and 39 controls) having missing results.

### Clinical characteristics of men with prostate cancer

The clinical characteristics of men with prostate cancer, were as follows: 396 (36.2%) had a Gleason score of 8 and above; 552 (51.3%) had a PSA score of >20ng/ml; 233 (21.7%) had metastatic prostate cancer at diagnosis; and 67 (6.1%) had clinical T3 disease. The risk categories of the nonmetastatic cases were low-risk 119 (17.7%), intermediate risk 282 (41.8%), and 273 (40.5%) classified as high/very high-risk (Table 3).

**Table 3:** Clinical characteristics of men with prostate cancer.

Variables	Total (N = 1095)	Men with prostate Cancer n(%)
**Gleason scores**	**1095**	
Gleason 6		119 (10.8)
Gleason 7		580 (53.0)
Gleason 8–10		396 (36.2)
**Tumour T staging**	1095	
T1 (1a-1c)		572 (52.2)
T2 (2a-2c)		456 (41.7)
T3 (3a-3c)		67 (6.1)
**PSA categories**	1077	
<10ng/ml		293 (27.2)
10-20ng/ml		232 (21.5)
>20ng/ml		552 (51.3)
**Nonmetastatic disease**	674	
Low risk		119 (17.7)
Intermediate risk		282 (41.8)
High/very high-risk		273 (40.5)
**Metastatic disease**	1076	
Positive		233 (21.7)
Negative		674 (62.6)
Not reported		169 (15.7)

### Frequency of metabolic diseases

In the whole cohort, only 27.3% (n = 583) of men reported no comorbid metabolic diseases; 43.0% (n = 918) reported at least one metabolic disease; 24.1% (n = 514) reported two metabolic diseases; 5.3% (n = 114) reported three metabolic diseases; and 0.3% (n = 7) reported four metabolic diseases. Cases had a significantly greater frequency of two or more metabolic diseases than controls (p<0.001) (Table 4).

**Table 4:** Frequency of metabolic diseases* in men, overall and by case/control status.

Metabolic diseases, n	Total (N = 2136), n (%)	Men with prostate Cancer (n = 1095), n(%)	Men without prostate cancer (n = 1041), n(%)	p-value
0	583(27.3)	201(18.4)	382(36.7)	**< .001**
1	918(43.0)	468(42.7)	450(43.2)	
2	514(24.1)	341(31.1)	173(16.6)	
3	114(5.3)	80(7.3)	34(3.3)	
4	7(0.3)	5(0.5)	2(0.2)	

*Defined as obesity (body mass index ≥30 kg/m2), dyslipidaemia (total cholesterol >6.21 mmol/L or self-reported HL on medication), hypertension (blood pressure ≥140/≥90 mmHg or medication use), hyperglycaemia (fasting glucose >5.5 mmol/L self-reported DM on medication)

### Frequency of at least one metabolic disease, depression, and HIV in men

As shown in Table 4, in the total cohort, the majority of men (66.2%, n = 1408) reported at least one metabolic disease, had a negative depression screen, and tested negative for HIV; 56.3% (n = 793) of this group were men with prostate cancer. Only 6.5% of men had at least one metabolic disease, tested positive for HIV and had a negative depression screen. Men without any comorbid conditions (no metabolic diseases, negative depression screen, and negative HIV test) comprised only 23.6% of the total cohort (n = 503), with the majority being in the control group (67.2%, n = 338). The prevalence of metabolic diseases and HIV-positive status was significantly higher among cases than in controls (p<0.001), and the prevalence of depression for the whole cohort was extremely low (Table 5).

**Table 5:** Frequency of at least one metabolic disease, depression, or HIV in men, overall and by case/control status.

At least one MD, D, HIV	Total (N = 2127), n (%)	Prostate Cancer (N = 1090), n (%)	Controls (N = 1037), n (%)	p-value
MD(+), D(-), HIV(-)	1408(66.2)	793(56.3)	615(43.7)	< .001
MD(+), D(-), HIV(+)	138(6.5)	96(69.6)	42(30.4)	
MD(+), D(+), HIV(-)	1(0.05)	1(100.0)	0(0.00)	
MD(-), D(-), HIV(+)	76(3.6)	35(46.1)	41(53.9)	
MD(+), D(+), HIV(+)	1(0.05)	0(0.00)	1(100.0)	
MD(-), D(-), HIV(-)	503(23.6)	165(32.8)	338(67.2)	

MD = Metabolic disease; D = depression and HIV = Human immunodeficiency virus

### Self-reported versus measured proportions of hypertension and high fasting blood sugar/diabetes

As shown in Tables 1 and 2, the proportion of men with measured impaired fasting glucose (45.4% and 36.3% respectively among cases and controls) was much greater than the proportion who self-reported being on treatment for diabetes (16.7% and 18.9% respectively among cases and controls). A high proportion of men in our cohort reported being on treatment for hypertension (66.3% and 48.7% respectively among cases and controls), yet anthropometric measurements in the clinic setting revealed a high rate of elevated blood pressure (57.5% of cases and 53.5% of controls).

### Factors associated with metastatic prostate cancer at diagnosis

We examined the factors associated with metastatic prostate cancer using stepwise multivariate models. In the socio-demographic characteristics model (model 1), older age was associated with metastatic prostate cancer at presentation (OR = 1.04 CI:1.02–1.07), whilst having secondary and or tertiary education appeared to be protective, although this was not significant (OR = 0.91 CI: 0.61–1.34)

In Model 2 we examined the association of comorbid metabolic diseases with metastatic prostate cancer at presentation and found that being obese (OR = 0.60 CI:0.37–0.97) was protective, compared to being overweight, normal weight or underweight. Furthermore, having hypertension as compared to being normotensive, also had a protective effect against metastatic prostate cancer (OR = 0.63 CL:0.42–0.92). Having dyslipidaemia (HDL <1.19 mmol/L), being diabetic and being HIV positive were not associated with metastatic prostate cancer (see Table 6).

**Table 6:** Multiple logistic regression models of factors associated with bone scan positive (metastatic) prostate cancer stage at diagnosis.

	Model 1, sociodemographic OR (95% CI)	Model 2, multimorbidities OR (95% CI)	Model 3. Sociodemographic + multimorbidities OR (95% CI)
**Sociodemographic**			
Age (years), mean (SD)	1.04(1.02–1.07)**		1.04(1.02–1.07)**
**Level of education**			
Primary education and below	**Reference**		**Reference**
Secondary and some tertiary education	0.91(0.61–1.34)		0.93(0.63–1.39)
**Communicable and non-communicable disease morbidities**			
**Obesity**			
No		**Reference**	Reference
Yes		0.60(0.37–0.97)*	0.63(0.39–1.02)
**Dyslipidaemia (High-density lipoprotein (HDL) in mmol/L**			
No		**Reference**	Reference
Yes		0.71(0.46–1.08)	0.72(0.47–1.11)
**Hypertension**			
No		**Reference**	Reference
Yes		0.63(0.42–0.92)*	0.55(0.37–0.83)**
**Fasting glucose**			
No		**Reference**	**Reference**
Yes		1.42(0.74–2.74)	1.57(0.81–3.07)
**HIV status**			
Positive		**Reference**	**Reference**
Negative		1.47(0.81–2.65)	1.12(0.61–2.07)
*Model Parameters*			
*AIC*	*747*.*52*	*725*.*28*	*715*.*11*
*BIC*	*761*.*61*	*753*.*24*	*752*.*40*

**p value < .005;

*p value < .05;

AIC = Akaike Information Criterion BIC = Bayesian Information Criterion for probabilistic models

In the fully adjusted model 3, older age remained associated with metastatic prostate cancer (OR = 1.04 CI:1.02–1.07) and hypertension (OR = 0.55 CI:0.37–0.83) continued to be protective (see Table 6).

### Factors associated with high/very high-risk non-metastatic prostate cancer at diagnosis

In a model restricted to men with non-metastatic prostate cancer at diagnosis, we evaluated the factors associated with high/very high-risk versus low/intermediate risk prostate cancer, using a stage cT3a, Gleason score of 8–10 independent of PSA values, or PSA of >20 ng/mL, and cT3b, cT3c or T4 disease as the cut-off to define the high/very high-risk non-metastatic disease. Older age was associated with high/very high-risk non-metastatic prostate cancer (OR = 1.03 CI:1.01–1.05), whilst having dyslipidaemia (HDL <1.19 mmol/L) had a protective effect (OR = 0.73 CI:0.54–0.98) (Table 7).

**Table 7:** Multiple logistic regression models of factors associated with advanced risk non-metastatic prostate cancer (PSA>20ng/ml at diagnosis)^a^.

	Model 1, sociodemographic OR (95% CI)	Model 2, multimorbidities OR (95% CI)	Model 3. Sociodemographic + multimorbidities OR (95% CI)
**Sociodemographic**			
Age (years), mean (SD)	1.03(1.02–1.05)**		1.03(1.01–1.05)**
**Level of education**			
Primary education and below	**Reference**		**Reference**
Secondary and some tertiary education	1.04(0.81–1.33)		1.06(0.82–1.37)
**Communicable and non-communicable disease morbidities**			
**Obesity**			
No		**Reference**	Reference
Yes		0.90(0.67–1.20)	0.94(0.70–1.27)
**Dyslipidaemia (High-density lipoprotein (HDL)** <1.19 mmol/)			
No		**Reference**	Reference
Yes		0.74(0.56–0.99)*	0.73(0.54–0.98)*
**Hypertension**			
No		**Reference**	Reference
Yes		0.96(0.74–1.26)	0.88(0.67–1.16)
**Fasting glucose**			
No		**Reference**	**Reference**
Yes		0.96(0.59–1.54)	1.03(063–1.67)
**HIV status**			
Positive		**Reference**	**Reference**
Negative		0.89(062–1.27)	0.73(0.50–1.06)
*Model Parameters*			
*AIC*	*1484*.*19*	*1442*.*80*	*1430*.*10*
*BIC*	*1499*.*13*	*1472*.*46*	*1469*.*65*

**p value < .005;

*p value < .05;

^a^This was used to classify locally advanced disease for non-metastatic cases only;

AIC = Akaike Information Criterion BIC = Bayesian Information Criterion for probabilistic models

## Discussion

In this hospital-based case-control study, we described the multimorbidity burden among African men in Soweto, SA with and without prostate cancer and determined the factors associated with metastatic and high/very high-risk non-metastatic prostate cancer at diagnosis. We found that more than 70.0% of participants had a metabolic disease, which was defined as having one or more of the following conditions: obesity (as measured by BMI), dyslipidaemia (self-reported and on treatment), hypertension (self-reported and on treatment) or diabetes/hyperglycaemia (self-reported and on treatment). Furthermore, we found that men with prostate cancer had a significantly greater prevalence of metabolic diseases than controls. Several factors such as dietary intake, alcohol and smoking and lack of physical activity might explain this high prevalence of metabolic diseases in both men with and without prostate cancer. Twelve percent (n = 247) of our cohort were HIV positive, with men with prostate cancer accounting for about 60.0% of this group, highlighting the overlapping burden of non-communicable and chronic diseases in the Soweto setting.

Comparing self-report with measured data for hypertension and diabetes, the burden of impaired glucose metabolism/diabetes appears vastly under-reported and a high proportion of hypertensive patients on treatment appear not to have well-controlled blood pressure. This finding is concerning for under-diagnosis and under-treatment of hypertension and diabetes in the SA setting and mirrors our prior findings from a Soweto case-control breast cancer cohort study [23]. There is underreporting or underestimation when self-reported as compared to actual measurements and this might be related to the low educational level among our cohort, where more than half (51.5%) of the men had primary education and below. It may also reflect the poor clinical management of common lifestyle-associated metabolic diseases in our public health setting. Therefore, carrying out and improving actual measurement rates in hospitals will likely foster the adoption of best practices, thus further improving clinical outcomes.

Overall, older age was associated with metastatic and high/very high-risk non-metastatic prostate cancer. Our finding that older age is associated with more advanced prostate cancer at diagnosis is in keeping with previously published literature [24, 25]. Studies of predominantly Caucasian men have also reported that older men (>60 years) are more likely to have treatment-refractory prostate cancer and may have shorter survival compared to younger men [2, 26]. A survival analysis of our cohort is pending and will provide much-needed prostate outcomes data for men of African descent living in SA.

We found a high prevalence of metabolic disease among men of African descent in Soweto, with a significantly higher burden among those with prostate cancer, consistent with findings from other Caucasian, African American and African populations [11, 27, 28]. An association between metabolic disease and advanced-stage prostate cancer has been reported in cohort studies from the USA and Europe [29, 30]. However, findings on the association between specific metabolic diseases and advanced-stage prostate cancer are conflicting. In our study, men with prostate cancer were more likely to be hypertensive and yet we found that hypertension was significantly protective against metastatic prostate cancer at diagnosis, compared to normotensive men. This protective effect against metastatic prostate cancer might be due to aggressive disease management among men with hypertension. Furthermore, this might reflect a more socioeconomically affluent group, more aware of prostate cancer and its symptoms as shown among women with breast cancer from higher socioeconomic households who conferred a 19% reduction in the odds of having advanced-stage breast cancer at diagnosis [23]. This finding is similar to what was reported in Sweden among a cohort of over 300 000 construction workers, where hypertension was associated with a decreased risk of incident prostate cancer (RR = 0.84 (95% CI, 0.76–0.91; p for trend <0.0001), although the reason for such protective effect was unclear [31]. However, conflicting findings have been reported for hypertension as a risk factor for prostate cancer [31–38]. A meta-analysis of 21 published studies indicated that patients with hypertension may be associated with an increased risk of prostate cancer (RR = 1.08, 95% CI:1.02–1.15, p = 0.014) [32].

Diabetes also seemed to be negatively associated with metastatic prostate cancer in our study, although this was not significant. In previously published literature, the association of diabetes with advanced-stage prostate cancer is similarly unclear [39–42]. In the United Kingdom, among a cohort of prostate cancer patients, they found type 2 diabetes to be associated with a 23% increased risk of prostate cancer mortality (HR 1.23, 95% CI 1.04–1.46) [39].

The large multi-centre case-control study of African men, which included participants from our cohort, found that overall obesity and central obesity were positively associated with D’Amargio intermediate-risk prostate cancer, but not with low and high-risk disease [11]. Patients with advanced disease likely lose weight.

Despite not being significant, there was an indication that being HIV positive might be associated with metastatic prostate cancer among our cohort (OR = 1.12 CI:0.61–2.07). This finding might be in sync with evidence that has shown that African men who are HIV positive have an associated increased risk of developing different HIV-associated malignancies [15, 43]. Whether HIV is associated with metastatic or high-risk non-metastatic prostate cancer needs to be explored further. In our setting, where the HIV burden is high, integrated prostate cancer screening services in antiretroviral therapy clinics, might be of benefit.

The very low depression levels reported for our cohort using the PHQ-9 depression score appear contradictory to other findings in South Africa and merit further examination. [44, 45].

### Study limitations

Our study findings may not be generalisable because this study was conducted at one hospital in Johannesburg, and participants who enrolled in this study almost exclusively resided in Soweto. Despite this limitation, our study has generated important information concerning the high burden of comorbidities among men with prostate cancer and highlights the need for improved chronic disease management in this population.

## Conclusion

The high prevalence of metabolic diseases and HIV among African men with prostate cancer in SA represents a public health concern. The treatment and management of prostate cancer in SA must evolve to better address the multimorbidities in this patient population. A holistic approach should be promoted through the effective implementation of coordinated care models.

## Supporting information

S1 Dataset (XLSX)
